# Perceptual Contiguity Does Not Modulate Matched-Case Identity-Priming Effects in Lexical Decision

**DOI:** 10.3390/brainsci13020336

**Published:** 2023-02-16

**Authors:** Marta Vergara-Martínez, María Fernández-López, Manuel Perea

**Affiliations:** 1 Interdisciplinary Structure of Reading Research (ERI.-Lectura), Universitat de València, 46010 Valencia, Spain; 2 Department of Developmental and Educational Psychology, Universitat de València, 46010 Valencia, Spain; 3 Department of Methodology of Behavioral Science, Universitat de València, 46010 Valencia, Spain; 4 Nebrija Research Center on Cognition (CINC), Universidad Nebrija, 28015 Madrid, Spain

**Keywords:** lexical decision, masked priming, orthographic processing, word recognition, color

## Abstract

In recent studies with the masked priming lexical decision task, matched-case identity-priming effects occur for nonwords but not for words (e.g., nonwords: ERTAR-ERTAR faster than ertar-ERTAR; words: ALTAR-ALTAR produces similar response times as altar-ALTAR). This dissociation is thought to result from lexical feedback influencing orthographic representations in word processing. As nonwords do not receive this feedback, bottom-up processing of prime–target integration leads to matched-case effects. However, the underlying mechanism of this effect in nonwords remains unclear. In this study, we added a color congruency manipulation across the prime and target in the matched-case identity-priming design. We aimed to determine whether the case effects originate at the early stages of prime–target perceptual integration or due to bottom-up activation of case-specific letter detectors. Results replicated the previous dissociation between words and nonwords regarding the matched-case identity effect. Additionally, we did not find any modulation of these effects by prime–target color congruency. These findings suggest that the locus of the matched-case identity effect is at an orthographic level of representation that encodes case information.

## 1. Introduction

Hierarchical, neurobiologically inspired models of letter and visual word recognition propose that perceptual parameters, such as font, size, case, or color, are rapidly disregarded when mapping visual input into abstract letter representations [1,2]. This implies that the presentation of words such as table, TABLE, or Table would activate, to a similar degree, the lexical representation corresponding to “table”.

This view is supported by evidence from masked priming experiments with word stimuli. For instance, Jacobs et al. [3] found that lexical decision times to a word such as TABLE were comparable regardless of whether it was briefly preceded by a matched-case identity prime (TABLE*)* or a mismatched-case identity prime (table). Critically, they also found that responses to a nonword such as SUGLE were faster when preceded by a matched-case identity prime (SUGLE) than when preceded by a mismatched-case identity prime (sugle). This dissociation has been replicated in other languages and tasks [4,5].

To further investigate this dissociation, Vergara-Martínez et al. [5] recorded participants’ event-related potentials (ERPs) in a masked priming lexical decision experiment. They found that the effect of matched-case identity priming for words and nonwords was present in an early, prelexical, perceptual component (N/P150), but it vanished for words in a component associated with the initial mapping to orthographic representations (N250) and in a component related to lexical-semantic activation (N400). However, for nonwords, the difference was still present in both the N250 and N400 components (see also [6] for the same pattern). This pattern suggests that lexical feedback overrides the differences arising at perceptual processing for mismatched-case identity primes in words, but not in nonwords.

The dissociation between words and nonwords regarding matched-case identity priming in behavioral and electrophysiological measures with the lexical decision task presents a significant challenge for models that assume a bottom-up mapping from visual features to abstract letter/word representations [1,2]. These models would predict similar responses for pairs such as table-TABLE versus TABLE-TABLE and sugle-SUGLE versus SUGLE-SUGLE. However, lexical-decision ERP findings from Vergara-Martínez et al.’s [5] and Gutierrez-Sigut et al.’s [6] experiments suggest that, for words, lexical feedback overrides the differences arising at perceptual processing for mismatched-case identity primes (see [7] for a review of bottom-up vs. interactive models of visual word recognition)

The underlying reason for the matched-case identity-priming effect in nonword responses is still unclear. One possibility is that, without top-down feedback, nonword prime processing leads to bottom-up activation of case-specific letter detectors. If the nonword target maps to the same detectors as the prime (e.g., as in SUGLE–SUGLE), evidence for the decision will accumulate faster than if the target maps to different detectors (e.g., sugle–SUGLE), resulting in faster reaction times.

Another interpretation of the dissociation between words and nonwords regarding the matched-case identity-priming effect is related to the nature of the masked priming technique. Mismatched-case manipulation may disrupt the integration of prime and target into a “unique” perceptual object, as evidenced by the early impact of the matched-case manipulation for both words and nonwords in the ERP measures (N/P150 effects) [5]. Later in processing, around 200 ms, and only for words, lexical feedback plays a role in mapping orthographic units onto more abstract representations. In contrast, for nonwords, the early-stage disruption of prime–target integration may also impact the reaction times in the lexical decision task.

To further investigate this interpretation, the present study includes a manipulation that may facilitate the integration of prime and target into a unique perceptual instance at early processing stages: color. The rationale is that since color can be used as a grouping feature, it can help define a perceptual object, so that targets and matching color primes can be easily integrated into a single category. Indeed, according to object file theory, different object files result from the feature-integration process in visual short-term memory [8,9]. In this context, color may help produce a specific grouping of the elements that constitute the objects depending on whether these elements share this feature. If we transfer this reasoning to a masked repetition priming scenario, a change in one feature that defines the object identity (color) across prime and target will lead to updating the previous file to such a degree that the novel object is perceived as a different one. In contrast, if no change occurs between tokens of the same type, file updating converges into the same object file, leading to object constancy and continuity. Integrating features into new object files would lead to slower reaction times than the result of combining the features into the same object file.

Thus, in the context of a perceptual account of matched-case identity-priming effects, using different colors across the prime and target would induce a clearer segregation of visual features into two separate entities. Keep in mind that the effect of matched-case identity priming starts in the very early (perceptual) stages of stimulus processing (less than 100 ms) (see [5]). A color mismatch across prime and target might enhance the matched-case identity effects obtained in nonword stimuli relative to a color match across prime and target pairs. Therefore, if color facilitates the integration of prime and target into a unique perceptual instance, matched-case identity-priming effects would be reduced for nonwords when color is used as a grouping feature.

In the present study, we aimed to examine the effect of color match on prime–target integration in a lexical decision task. To this end, we utilized an orthogonal design to manipulate three relevant variables: matched-case identity priming, lexicality, and matched-color identity priming (e.g., altar–ALTAR vs. altar–ALTAR and ALTAR–ALTAR vs. ALTAR–ALTAR; mabar–MABAR vs. mabar–MABAR and MABAR–MABAR vs. MABAR–MABAR [mabar is a nonword]). For comparison purposes, we employed the same set of stimuli as Vergara-Martínez et al. [6]. We chose green and red because previous research has shown that these colors are very effective as perceptual cues during reading (see [10,11]).

Our predictions for this study were as follows: If masked identity priming in a lexical decision task is based on the integration of abstract representations (see [12]), lexical decision responses would be similar for matched-color and mismatched-color identity pairs. Therefore, the dissociation between words and nonwords regarding the matched-case identity-priming effect would still be observed regardless of the matched-color manipulation. On the other hand, if color is an effective perceptual cue in binding visual features into an independent/unique perceptual object in the first stages of word processing, the magnitude of matched-case priming effects would be greater for mabar-MABAR (i.e., same color) than for mabar-MABAR (i.e., different colors). This latter outcome would challenge the interpretation of masked priming effects on the lexical decision task that focused on the role of abstract letter/word representations. Conversely, if the visual perceptual manipulations fail to modulate the matched-case identity-priming effect, an interpretation of the matched-case effects in terms of perceptual contiguity across prime and target would need to be discarded.

## 2. Materials and Methods

### 2.1. Participants

A group of twenty-four undergraduate and graduate students of the University of Valencia (21 women) took part in the experiment. This allowed us to obtain a similar number of observations as in previous examinations of the case-mismatch dissociation [4,5]. All of the participants were native speakers of Spanish with normal/corrected-to-normal vision and no history of reading disorders. Before starting the experiment, all participants signed an informed consent form.

### 2.2. Materials

We selected the same set of stimuli as in the Vergara-Martínez et al. experiments [5]: 160 five-letter words from the EsPal database [13] and 160 nonwords created with Wuggy [14]. For the word stimuli, the mean frequency was 4.3 (range: 3.04–5.32) and the mean OLD20 was 1.4 (range: 1.00–2.30) in EsPal [13]. For each participant, the targets were always presented in uppercase, either in red or green (e.g., ALTAR or ALTAR; MABAR or MABAR), and were preceded by: (1) an identity prime matched in color and case with the target (total-match identity condition; e.g., ALTAR–ALTAR; MABAR–MABAR); (2) an identity prime that was matched in letter-case with the target, but not in color (color-mismatched identity condition; e.g., ALTAR–ALTER; MABAR–MABAR); (3) an identity prime that was matched in color with the target, but unmatched in case (case-mismatched identity condition; e.g., altar–ALTAR; mabar–MABAR); and (4) an identity prime that was unmatched in both color and letter-case (total-mismatched identity condition; e.g., altar–ALTAR; mabar–MABAR). For control purposes, we created another eight conditions with the same characteristics as the previous but using unrelated primes instead of identity primes: four conditions with an unrelated word prime (e.g., TÚNEL–ALTAR; QUESO–MABAR) and another four with an unrelated nonword prime (TAROR–ALTAR; BIUTO–MABAR). Counterbalanced lists were created so that each target stimulus was rotated across the different conditions. The lists of pairs are available in the OSF link given in the Data Availability Statement.

### 2.3. Procedure

Each participant was tested in groups of up to four individuals in a quiet room. The presentation of the sequence of stimuli and registration of participants’ responses was carried out using DMDX software [15]. As in the Vergara-Martínez et al. [5] experiment and the Perea et al. [4] experiments, in each trial, a forward pattern mask (i.e., a series of # in black) was presented for 500 ms. Next, the prime was shown for 33 ms (i.e., two refresh cycles at 60 Hz), which was replaced with a 16 ms pattern mask (i.e., one refresh cycle). Then, the target stimulus was presented in the same spatial location as the prime until the participant responded or 2 s had elapsed. All prime and target stimuli were presented in a colored monospaced font (Courier New 14 pt) on a white background. The colors were either red (RGB:208-0-0) or green (RGB:0-131-37). Participants performed a lexical decision task: they had to decide as accurately and rapidly as possible whether or not the stimulus was a Spanish word, and pressed one of the response buttons (YES/NO). The instructions stressed both speed and precision of the responses. Sixteen practice trials preceded the 320 experimental trials. Participants did not receive feedback on reaction times or error rates during the experiment. The session lasted for around 15 min.

### 2.4. Statistical Analyses

To examine whether color congruency of the matched- and mismatched-case prime–target pairs modulates masked identity priming, we examined the identity pairs (3840 observations; i.e., total-matched, color-unmatched, case-unmatched, and total-unmatched). Error responses (156 observations) and extremely short RTs (less than 250 ms: 2 observations) were excluded from the latency analyses—as indicated above, the deadline to respond was set to 2 s (the computer program categorized “no responses” as errors).

For the inferential analyses, we used Bayesian linear mixed effects with the brms package [16] in the R environment [17]. The design had three fixed factors: target lexicality (word [coded as −0.5], nonword [coded as −0.5]), prime case (matched [−0.5], mismatched [0.5]), and prime color (matched [−0.5], mismatched [0.5]). For both latency and accuracy data, we fit the maximal random-effects model:

Dep.Variable (RT or accuracy) ~ lexicality * color * case + (1 + lexicality * color * case | subject) + (1 + color * case | item)

To model the response times, we used the ex-Gaussian as the underlying distribution (i.e., a convolution of the normal and exponential distribution [18]), whereas to model the accuracy data, we used the Bernoulli distribution (where 1 = correct response and 0 = incorrect response). For each model, we conducted 5000 iterations (1000 for warm-up) with four chains. All values of 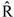 were 1.00 (i.e., the four chains converged to the same point).

## 3. Results

The mean RTs for the correct responses and the accuracy in each experimental condition are presented in Table 1.

**Figure 1:**
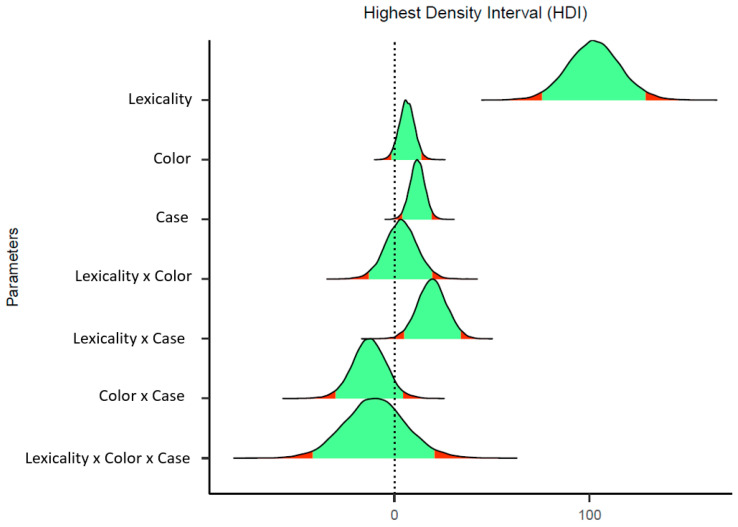
Posterior distributions from the Bayesian linear mixed-effects models for each of the effects. The red area reflects the parameter values beyond the 95% Credible Intervals.

The estimates of each parameter of the model, together with the 95% credible intervals, are given in Table 2 An effect was interpreted as significant when its 95% credible interval did not overlap zero. The posterior distribution of each effect is presented in Figure 1. In the case of a significant interaction, we computed simple effects tests using the 95% Highest Posterior Density Interval (95% HPD) with the emmeans package [19].

### 3.1. Latency Analyses

As expected, we found the typical lexicality effect: response times were faster for word targets than for nonword targets (624 vs. 769 ms, respectively; *b* = 102.29, SE = 13.48 95% CrI [75.93, 129.04]). Furthermore, as in previous research, we found the expected interaction between lexicality and case (*b* = 19.35, SE = 7.37, 95% CrI [4.75, 33.83]). This interaction reflected that, for nonword targets, responses were faster in the matched-case than in the mismatched-case condition (756 vs. 782 ms, respectively; estimate: −21.42; 95% CrI [−32.4, −10.91]), whereas for word targets, there were no signs of an effect (estimate: −2.13 ms; 95% HPD [−11.8, 8.05]). There was some evidence of a small effect of color mismatch (*b* = 6.38, SE = 3.94, 95% CrI [−1.33, 14.05]), but its credible interval crossed zero. Critically, no other estimates showed evidence of an effect (see Figure 1 for the posterior distributions of each parameter).

### 3.2. Accuracy Analyses

We did not find evidence of any effects of interactions—note that accuracy was close to ceiling in all conditions (see Table 1).

## 4. Discussion

A masked identity-priming experiment utilizing the lexical decision task was conducted to determine whether prime–target color congruency (e.g., ALTAR–ALTAR, MABAR–MABAR vs. ALTAR–ALTAR, MABAR–MABAR) had an impact on identity priming in a similar manner as case-match between prime and target (e.g., for nonword targets, MABAR–MABAR produces faster latencies than mabar–MABAR) [3,4,5]. The results revealed a matched-case congruency advantage for nonwords (a 26-ms difference) that was not present for word targets, replicating previous findings. Notably, prime–target color congruency did not interact with the masked identity-priming effect (see Figure 1 for the posterior distributions).

Thus, the results of the present experiment revealed a matched-case congruency advantage for nonword targets but not for word targets. This outcome, which replicates previous findings, suggests that the retrieval of abstract letter representations in the lexical decision task depends on activating lexical entries. In the absence of lexical feedback, such as nonword stimuli, matched-case identity priming persists. As stated in the Introduction, we aimed to examine the underlying explanation for this effect in nonword stimuli. Previous research has suggested that in the absence of lexical feedback, nonword prime processing might integrate case-specific letter detectors [4,5]. If the subsequent nonword target maps the same case-specific detectors, evidence towards a decision will accumulate faster, resulting in faster response times. However, an alternative explanation is that this effect could be partially due to perceptual contiguity between the prime and target.

To differentiate between these two explanations, we included a manipulation of color congruity across the prime and target. Perceptual features, such as shape and color, would aid in forming an object file at a perceptual level [20,21]. Moreover, research has shown that even task-irrelevant information, such as color in a lexical decision task, can lead to masked priming effects [22]. Therefore, if matched-case identity-priming effects in nonwords were based on perceptual contiguity, we would have expected to find larger effects when the prime and target matched in case and color. However, this was not the pattern observed in our experiment. Instead, we found a matched-case identity-priming effect for nonwords regardless of color congruity, thus strengthening the explanation that this effect is due to the activation of specific case letter detectors. This interpretation is further supported by the results of Perea et al.’s [4] Experiment 2, which found that the matched-case identity-priming effect in nonwords was not a function of the visual similarity between the allographic versions of the letters. Thus, we can conclude that this effect is not merely due to feature overlap or perceptual contiguity but rather takes place at an orthographic level of representation that still encodes letter-case information.

The present pattern of results is partially consistent with Dehaene et al.’s [1] LCD model of visual word recognition, where perceptual characteristics of letters (i.e., color) and words are dismissed along the way to meaning. Specifically, this model proposes a hierarchy of increasingly larger and more abstract neural computations on the stimulus, from primary (e.g., shape, color) to complex visual features, then to abstract letters, letter sequences, and words along the left ventral pathway [1,23,24]. At earlier processing stages, visual information for letter strings such as snow, SNOW, or the nonword nigelo or NIGELO activates different shape-fragment detectors, which are also sensitive to letter-case (i.e., the locus of our present results). Higher in the hierarchy, at later stages of processing, different neural populations (abstract letter detectors) map letter shapes into abstract units free from size, font, case, and orientation information [25]. While the LCD model posits that these abstract units play a central role in visual word recognition, the present experiment has shown that these operations may not be fully feedforward or automatic. The dissociation of matched-case identity-priming effects between words and nonwords in lexical decision tasks poses some problems for the feedforward nature of Dehaene et al.’s LCD model; instead, our findings fit better with fully interactive models of visual word recognition [4,5,6,7]. The automatic nature of these operations was also challenged by a study conducted by Perea et al. [26], where the authors employed a masked priming task that taps early perceptual pre-lexical processes and minimized top-down lexical processing (namely, a same–different task). With this task, Perea et al. [24] found faster responses for matched-case identity not only for nonwords (SUGLE–SUGLE faster than sugle–SUGLE) but also for words (TABLE–TABLE faster than table–TABLE).

We acknowledge that, as in any behavioral study, the findings of the present paper do not provide information regarding the temporal dynamics of prime–target processing. Future research should be conducted to provide more insight on this matter. Based on previous research using event-related potentials [5,25,27], it is likely that differences will be observed in the waveforms when the primes and targets are matched vs. mismatched in color in the very early time windows (i.e., those related to perceptual processing, e.g., N/P150). However, these differences may not persist in subsequent time windows more associated with orthographic processing, nor are they sizeable in response times, as demonstrated in the present study. Another avenue for future research would be to examine whether the effects of color congruency may be more pronounced in a task where the color of the stimuli (e.g., green vs. red) holds more meaning, such as in an affective decision task, and whether these effects are stronger for longer stimuli.

In conclusion, our findings suggest that an explanation of matched-case identity priming on nonwords based on perceptual contiguity in the lexical decision task can be disregarded. The color match between the prime and target did not affect the typical pattern of effects observed in previous lexical decision experiments, which revealed a dissociation of matched-case identity-priming effects for words and nonwords. Therefore, the effects observed here reflect the interactive dynamics of bottom-up and top-down activity in visual word recognition (see [6,28] for similarities and differences of this interplay in deaf readers). In the attentional sensitization model of unconscious cognition, task-relevant information is prioritized and task-irrelevant influences are attenuated at both a conscious and unconscious level [29,30]. In the lexical decision task, attention to task-relevant information (i.e., letter identification) seems to modulate lower-level cognitive operations such as the perceptual integration of the prime and target [5,22]. Thus, this task may prioritize cognitive resources towards lexical-semantic processing, and irrelevant information, such as the color of the letters, may be rapidly discarded in higher-level processing.

## Figures and Tables

**Table 1:** Averages of the response times (in ms) and accuracy in each of the experimental conditions.

	Case-Matched		Case-Mismatched	
	Color-Matched	Color-Mismatched	Color-Matched	Color-Mismatched
	ALTAR–ALTAR	ALTAR–ALTAR	altar–ALTAR	altar–ALTAR
Word	622 (0.979)	631 (0.975)	618 (0.960)	627 (0.969)
	MABAR–MABAR	MABAR–MABAR	mabar–MABAR	mabar–MABAR
Nonword	743 (0.952)	769 (0.940)	778 (0.940)	785 (0.960)

**Table 2:** Estimates from the Bayesian linear mixed-effects models for each of the critical conditions in the latency and accuracy analyses of the experiment.

	Estimate	Est. Error	95% Credible Interval
Latency data			
**Lexicality**	**102.29**	**13.48**	**75.93–129.04**
Color	6.38	3.94	−1.33–14.05
**Case**	**11.76**	**3.83**	**4.22–19.25**
Lexicality × Color	3.42	8.25	−12.95–19.61
**Lexicality × Case**	**19.35**	**7.37**	**4.75–33.83**
Color × Case	−12.88	8.72	−30.35–4.23
Lexicality × Color × Case	−9.91	15.80	−40.83–21.80
Accuracy data			
Lexicality	−0.62	0.53	−1.66–0.44
Color	0.08	0.57	−1.00–1.23
Case	−0.79	0.47	−1.73–0.11
Lexicality × Color	−0.12	0.63	−1.34–1.14
Lexicality × Case	0.63	0.55	−0.45–1.74
Color × Case	0.88	0.73	−0.48–2.39
Lexicality × Color × Case	0.19	0.81	−1.39–1.80

Note: the parameters in **bold** indicate that the 95% credible interval did not cross zero.

## Data Availability

The stimuli, data, scripts, and results are available at the following link: https://osf.io/j63e4/?view_only=6a9ec9dcafb942008ea446826ea465d3 (accessed on 16 January 2023).
